# Resection of a giant thoracic solitary fibrous tumor treated with preoperative arterial coiling followed by a double-level thoracotomy

**DOI:** 10.1093/jscr/rjad008

**Published:** 2023-01-19

**Authors:** Jessica Cox, Haley Leesley, Abe DeAnda, Diana Palacio Uran, Scott Lick

**Affiliations:** School of Medicine, University of Texas Medical Branch, Galveston, TX 77550, USA; Department of Cardiovascular and Thoracic Surgery, University of Texas Medical Branch, Galveston, TX 77550, USA; Department of Cardiovascular and Thoracic Surgery, University of Texas Medical Branch, Galveston, TX 77550, USA; Department of Radiology, University of Texas Medical Branch, Galveston, TX 77550, USA; Department of Cardiovascular and Thoracic Surgery, University of Texas Medical Branch, Galveston, TX 77550, USA

## Abstract

Solitary fibrous tumor (SFT) are rare pleura neoplasms often localized to middle or inferior hemithorax. A middle-aged woman presents to the emergency department following a motor vehicle accident, the computed tomography scan revealed a giant tumor occupying the entire left pleural cavity with a complete collapse of the left lung and substantial right deviation of heart and mediastinum. Using preoperative arterial coiling followed by a double-level thoracotomy we successfully resected the giant tumor. The SFT weighed ~10 lbs. At 2-month follow-up visit patient reports mild discomfort during strenuous movement/heavy lifting but denies any shortness of breath.

## INTRODUCTION

Solitary fibrous tumors (SFT) are defined as mesenchymal neoplasms of submesothelial origin with fibroblastic differentiation, typically benign in nature [1]. SFTs of the pleura typically have an indolent clinical course, are asymptomatic and found incidentally. When they do become symptomatic, symptoms can include shortness of breath, pain and weight loss [1, 6]. The tumors have intrinsic and extrinsic neovascularization that can be visualized with computed tomography (CT) scan of adjacent structures is a characteristic finding of SFT on CT scans [2, 6]. The standard of care for SFT is surgical resection [2].

We present the case of a giant pleuro-parenchymal SFT treated with preoperative arterial coiling followed by resection by a double-level thoracotomy.

## CASE REPORT

**Figure 1:**
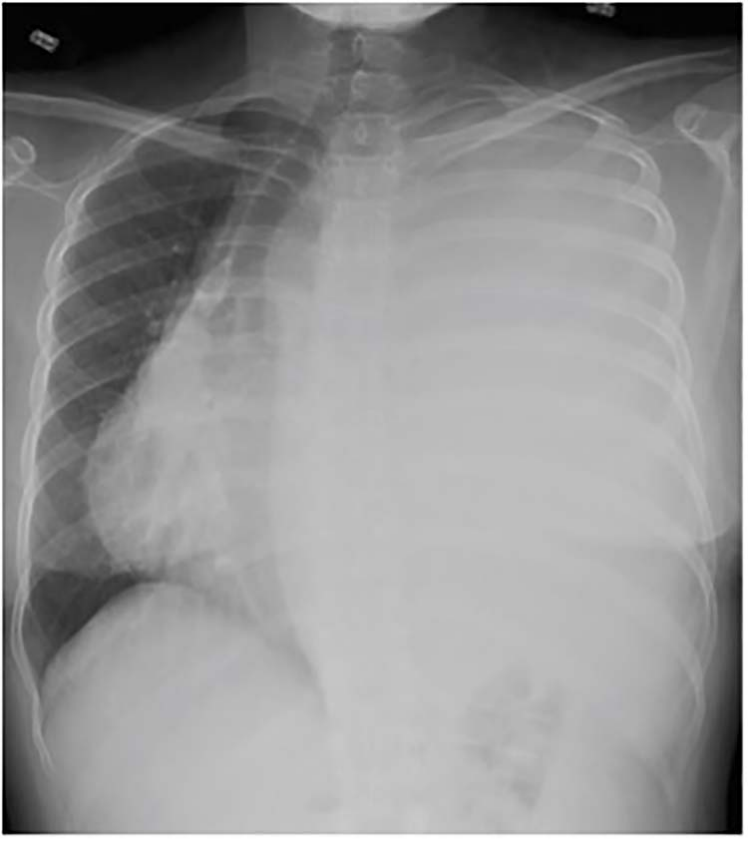
Frontal chest radiograph shows complete opacification of left hemithorax with significant mass effect.

A 33-year-old woman presented to the emergency department following a motor vehicle accident. Imaging showed a large heterogenous intermediate density mass filling and expanding the left hemithorax, causing a complete collapse of the left lung and substantial right deviation of heart and mediastinum (Fig. 1). The patient complained of mild exertional dyspnea that had worsen over the past 6 months. Physical exam was unremarkable aside from decreased breath sounds on left side. Percutaneous core needle biopsy revealed spindle cells along with immunostatins consistent with SFT. Upon later questioning, the patient noted progressive anorexia with a 15 lb weight loss over the last 4 months and occasional dyspnea.

**Figure 2:**
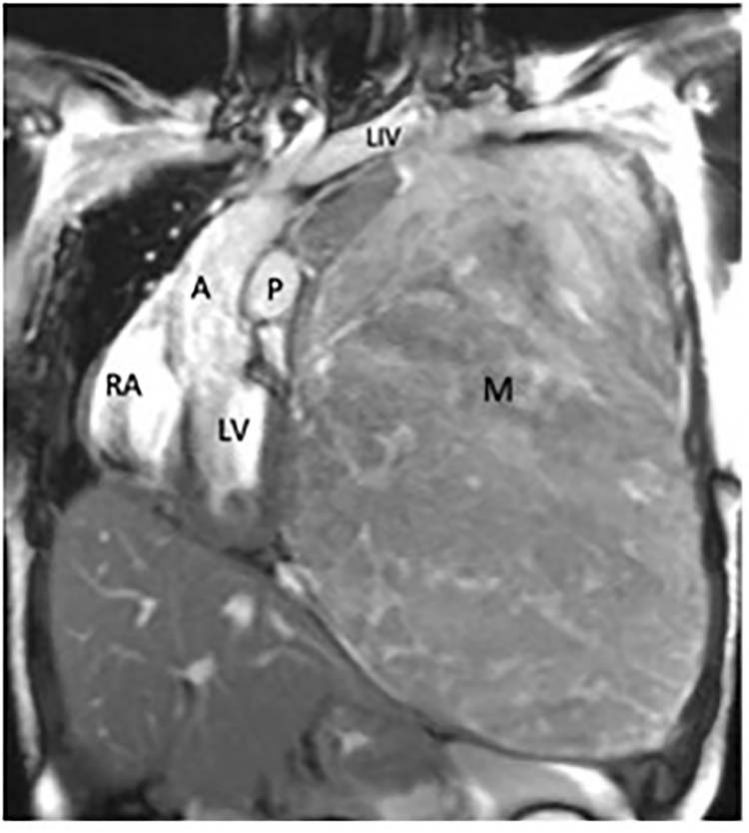
Coronal magnetic resonance image, showing the large heterogeneous mass occupying the left hemithorax. Note severe displacement of the heart and vasculature as well as complete atelectasis of the lung (L), left ventricle (LV), right atrium (RA), aorta (A), pulmonary trunk (P).

**Figure 3:**
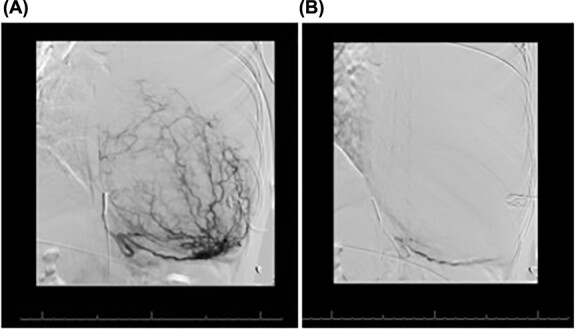
(**A**) Conventional angiogram engaging the left inferior phrenic artery, which perfuses much of the mass. (**B**) Postembolization results, demonstrating marked decrease in the mass blood supply.

Magnetic resonance imaging showed no invasion of chest wall or visceral structures, consistent with resectability (Fig. 2). To prepare for resection, thoracic aortography showed 75% of tumor arterial supply from left inferior phrenic artery, which was subsequently coiled with particles (500 μm and 700 μm embospheres) (Fig. 3A). Postembolization arteriogram demonstrated near complete cessation of anterior flow within this vessel and its branches (Fig. 3B).

**Figure 4:**
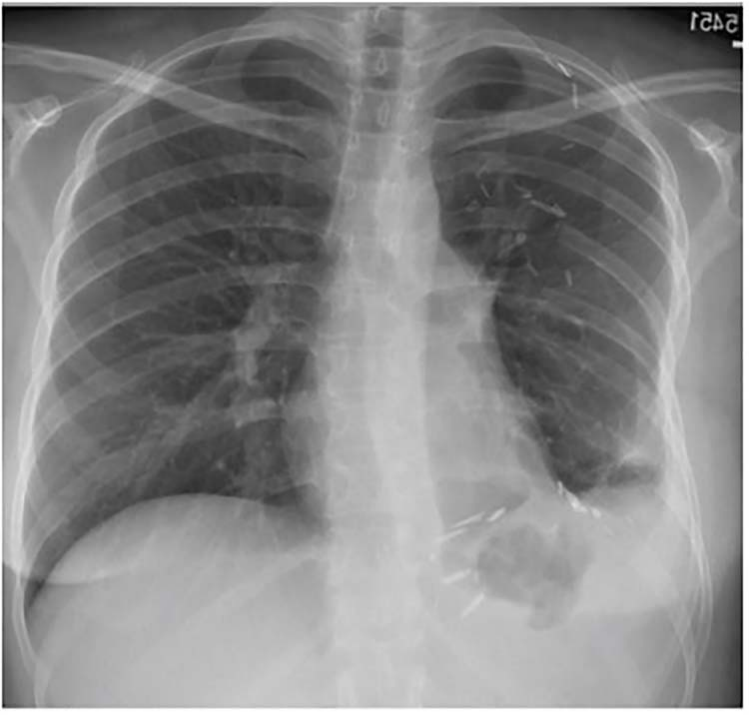
Frontal chest radiograph 2 months after resection with near-complete left lung expansion.

The following day a double thoracotomy was performed at the fifth and eighth intercostal space via single oblique skin incision, due to the expansion of the tumor from the left apex to the diaphragm. This allowed satisfactory exposure of the upper and lower aspects of the tumor, with the mass being delivered from the chest through the eighth interspace opening. The upper and lower lobes of the left lung were able to expand successfully. The tumor measured 22 × 26 × 11 cm, weighing ~10 lbs. The patient’s left lung expanded almost fully, and she was discharged one week post-op. At 2-month follow-up visit patient reports mild discomfort during strenuous movement/heavy lifting but denies any shortness of breath (Fig. 4).

The institutional review board waived the need for written consent 24 October 2022.

## DISCUSSION

SFTs were first described by Klemperer and Rabin in 1931. Although originally identified as rare primary pleural neoplasms of mesenchymal origin, immunohistochemical analysis has indicated these fibromas originate from the mesenchyme rather than mesothelial cells [3, 4]. SFTs are rare, representing <5% of all pleural tumors, and are typically benign [5]. The majority of SFTs arise from the visceral pleura with adhesions to adjacent pleural surfaces [6]. These tumors are typically localized to the middle and inferior hemithorax and visualized on CT scans as homogenous, well defined, non-invasive, lobular, soft tissue masses [7].

Because of the large size of this tumor, we pursued preoperative arterial occlusion, as operative approach to the vascular supply was expected to be difficult due to the exposure constraints of the ribcage and diaphragm. Preoperative angiography is beneficial for identifying and embolizing major feeding vessels [5]. With the extensive size of the tumor (22 × 26 × 11 cm), a double thoracotomy was indicated for proper resection, which allowed access to both ends of the tumor to free up adhesions.

These tumors are rare, and their sheer sizes within the constraints of the thorax make them difficult to resect. Preoperative arterial embolization has been reported as an adjunct, and double thoracotomy has also been reported for these tumors. We found the combination of these maneuvers to be very helpful in our case: the diaphragm surface required some blunt dissection, which was done without massive bleeding, and the double thoracotomy allowed good exposure to the apex, as well as an adequate opening for tumor delivery through the lower thoracotomy. We think that both embolization and double thoracotomy together allowed for safe removal of this challenging giant tumor.
